# Effects of Green and Gold Kiwifruit Varieties on Antioxidant Neuroprotective Potential in Pigs as a Model for Human Adults

**DOI:** 10.3390/nu16081097

**Published:** 2024-04-09

**Authors:** Alexander P. Kanon, Caroline Giezenaar, Nicole C. Roy, Isuri A. Jayawardana, Dominic Lomiwes, Carlos A. Montoya, Warren C. McNabb, Sharon J. Henare

**Affiliations:** 1 School of Health Sciences, College of Health, Massey University, Palmerston North 4442, New Zealand; a.kanon@massey.ac.nz; 2 Riddet Institute, Massey University, Te Ohu Rangahau Kai Facility, Palmerston North 4442, New Zealand; c.giezenaar@massey.ac.nz (C.G.); nicole.roy@otago.ac.nz (N.C.R.); isurijayawardana@yahoo.com (I.A.J.); carlos.montoya@agresearch.co.nz (C.A.M.); w.mcnabb@massey.ac.nz (W.C.M.); 3 Alpha-Massey Natural Nutraceutical Research Centre, Massey University, Palmerston North 4442, New Zealand; 4 Food Experience and Sensory Testing Laboratory, School of Food and Advanced Technology, Palmerston North 4410, New Zealand; 5 Department of Human Nutrition, University of Otago, Dunedin 9016, New Zealand; 6 High-Value Nutrition National Science Challenge, Auckland 1023, New Zealand; 7 Immune Health and Physical Performance, Nutrition and Health Group, The New Zealand Institute for Plant and Food Research Limited, Palmerston North 4410, New Zealand; dominic.lomiwes@plantandfood.co.nz; 8 Smart Foods and Bioproducts, AgResearch Ltd., Te Ohu Rangahau Kai Facility, Palmerston North 4442, New Zealand

**Keywords:** kiwifruit, antioxidant, neuroprotection, actinidin, brain regions, acetylcholinesterase

## Abstract

Kiwifruit (KF) has shown neuroprotective potential in cell-based and rodent models by augmenting the capacity of endogenous antioxidant systems. This study aimed to determine whether KF consumption modulates the antioxidant capacity of plasma and brain tissue in growing pigs. Eighteen male pigs were divided equally into three groups: (1) bread, (2) bread + *Actinidia deliciosa* cv. ‘Hayward’ (green-fleshed), and (3) bread + *A. chinensis* cv. ‘Hort16A’ (yellow-fleshed). Following consumption of the diets for eight days, plasma and brain tissue (brain stem, corpus striatum, hippocampus, and prefrontal cortex) were collected and measured for biomarkers of antioxidant capacity, enzyme activity, and protein expression assessments. Green KF significantly increased ferric-reducing antioxidant potential (FRAP) in plasma and all brain regions compared with the bread-only diet. Gold KF increased plasma ascorbate concentration and trended towards reducing acetylcholinesterase activity in the brain compared with the bread-only diet. Pearson correlation analysis revealed a significant positive correlation between FRAP in the brain stem, prefrontal cortex, and hippocampus with the total polyphenol concentration of dietary interventions. These findings provide exploratory evidence for the benefits of KF constituents in augmenting the brain’s antioxidant capacity that may support neurological homeostasis during oxidative stress.

## 1. Introduction

Numerous physiological and pathological processes increase the body’s reactive oxygen species (ROS) concentration, including physiological or emotional stress and sleep disturbances. Stressful conditions can result in the excessive production of ROS, overwhelming the body’s ability to neutralize them and leading to oxidative stress and damage. This process depletes endogenous antioxidants and disrupts the homeostatic mechanisms that maintain cellular health. The imbalance between pro-oxidants and antioxidants in the presence of ROS is a key factor in neuronal damage and the development of neurodegenerative diseases. Maintaining a delicate balance between these factors is essential for preserving cellular health and preventing oxidative damage in the brain [1]. The brain’s sensitivity to oxidative stress is attributed to its high energy demands, the abundance of lipids, and weak antioxidant capacity. This susceptibility to oxidative damage can cause neuronal impairment and functional decline through ROS-mediated brain tissue damage, ultimately increasing the risk of neurodegenerative diseases [2]. Specifically, ROS play a crucial role in this process by increasing the brain’s susceptibility to damage. Diet and nutrients have been shown to reduce these risks by enhancing the antioxidant system activities, thereby protecting from the deleterious effects of oxidative stress and ultimately leading to improved brain health [3,4,5].

Superoxide dismutase (SOD) is essential in reducing oxidative stress by converting ROS into hydrogen peroxide (H_2_O_2_), which is subsequently converted into inert molecules by other antioxidant enzymes. Malondialdehyde (MDA) is a marker of lipid oxidation, and excessive MDA induced by oxidative stress is associated with major depressive disorder [6]. Acetylcholinesterase (AChE) is the enzyme responsible for breaking down acetylcholine (ACh), and decreased levels of ACh are associated with neurological disorders. Likewise, increasing AChE activity is associated with neurological pathogenesis [7]. This has led to the development of therapeutics that target the treatment of these disorders by inhibiting or reducing brain AChE activity.

Kiwifruit (KF) has been shown to improve subjective mood [8,9] and sleep quality [10] in human clinical studies. Furthermore, studies have also shown that consumption of fresh KF may regulate antioxidant and inflammatory states [11,12,13]. KF is a rich source of vitamin C, vitamin E, and polyphenols, which serve as antioxidants, and it is hypothesized that this may be a mechanism by which KF improves mood [8] and sleep quality [10]. The majority of the before-mentioned clinical trials incorporating KF supplementation have employed a standardized serving size of the flesh from two fresh KF, adhering to established norms. Several in vitro studies have demonstrated that KF is neuroprotective through its efficacy in attenuating cellular ROS while increasing cell viability [14,15,16,17]. Rodent models have demonstrated the benefit of KF on brain health. A study of mice fed a high-fat diet (to induce stress) reported reduced oxidative stress in brain regions of mice supplemented with an extract of the KF *Actinidia arguta* (known for its high actinidin activity and content). The reduction in ROS reported in this study was concurrent with increased ACh, improved SOD activity, and reduced MDA and AChE activity in brain tissue [14]. In another study, Sprague Dawley rats with lead-induced cognitive dysfunction and neurodegeneration that were fed ‘Qinmei’ KF had higher hippocampal SOD and glutathione peroxidase (GPx) activities compared with those fed the placebo intervention [15].

While rodent models serve a valuable purpose, translating findings from these models to humans is constrained by significant physiological and metabolic differences. For instance, rodents exhibit a higher mass-specific metabolic rate, and their digestive physiology is less complex than that of humans. Additionally, it is important to comprehend the potential advantages of KF in healthy states, as rodent studies assessing the impact of KF have introduced an external stressor through diet or supplementation. Compared with rodents, the physiological function and brain anatomy of pigs share closer affinity to humans [18]. Moreover, pigs can consume KF in a manner like humans, whereas in rodent studies, the KF needs to be freeze-dried and ground, potentially resulting in a loss of some of its properties. Thus, employing pig models to investigate the effects of KF interventions on brain health outcomes may offer a more accurate reflection of these outcomes in the human brain than rodent models.

This study primarily explored the antioxidant effects of KF. The samples were collected as part of a larger study aiming to map the digestion of gluten and gluten-derived immunogenic peptides in the gastrointestinal tract of a pig model that was fed a meal containing gluten. The aim of this study was to evaluate the effect of consumption of green and gold KF interventions over one week, compared to a control diet without KF, on plasma and brain antioxidant levels in a healthy pig model. The distinguishing features between the two KF interventions are their micronutrient compositions and the presence of actinidin, a protease enzyme, in the green KF variety, which is absent in the tested gold variety. Actinidin is an enzyme needed for the cleavage of kiwellin (a minor protein in KF) into KiTH and kissper [19]. Kissper is suggested to form ion-channel-like pores by integrating itself into the phospholipid membrane [20] and may have anti-inflammatory properties [21]. This, in turn, could influence ROS. Whether these proteins are responsible for the beneficial effects in the brain has yet to be determined in vivo. We hypothesized that both KF interventions would improve both plasma and brain antioxidant markers, and the green KF variety may be more effective due to the presence of actinidin and extra micronutrients.

## 2. Materials and Methods

### 2.1. Animals and Treatments

Briefly, a total of 18 entire male pigs (*n* = 6 per diet; body weight (BW): 19.9 ± 0.5 kg, mean ± SE) of nine weeks of age were used in this study. Pigs were housed individually in metabolism crates (1.5 m × 1.5 m) in a room with a 12 h:12 h light/dark cycle and maintained at 22 ± 2 °C (Animal Processing Unit Massey University). Water was provided ad libitum. The Massey University Animal Ethics Committee, Palmerston North, New Zealand, approved all animal-related procedures (MUAEC protocol 20/46) [22]. All experiment stages, from group allocation to data analysis, were known to the researchers and technicians involved.

On arrival, pigs were fed the same commercial grower mix as provided on the farm. From the following day, experimental diets replaced this commercial diet gradually per day by 33% increments. Each pig received 90 g dry matter of feed per kilogram of metabolic BW per day (metabolic BW was calculated as = BW^0.75^) [23]. The pigs were provided with a solid meal portion size of 2:1 at 0900 and 1600 h, respectively. The pigs were adapted to their experimental diet for eight days, and on the final day were fasted and euthanized. Pigs were assigned to the following experimental diets:

- Soda bread only.
- Soda bread + gold KF (*Actinidia chinensis* cv. ‘Hort16A’).
- Soda bread + green KF (*Actinidia deliciosa* cv. ‘Hayward’).

To limit the digestive system’s exposure to proteins other than those derived from wheat, soda bread was employed in this study, as the focus was to evaluate the effect of KF on digestion of proteins originating from gluten. For the adaptation diets, pure gluten was introduced to reach the daily protein requirements. Before feeding the pigs, soda bread slices were cut into small pieces, and KF pulp was crushed manually before mixing it thoroughly with the bread. The fresh KF included in the diets comprised 19% of the daily dry matter intake, equivalent to two fresh KF eaten with a meal [23]. The actinidin activities were 27.08 ± 1.20 and 0.22 ± 0.15 U/g of dry matter for green and gold KF, respectively [22]. Average daily nutrient intake was calculated from when pigs were on experimental diets (Table 1). Commercially available vitamin and mineral premixes were added to the adaptation diets according to National Research Council [24] recommendations for the pigs’ daily nutritional requirements.

### 2.2. Euthanasia and Tissue Collection

On the final day of the experimental diet, pigs were euthanized 300 min after their final meal. This final meal was smaller than their adaptation meals to mimic a standard meal size of bread intake by humans, consisting of four slices of bread (178.2 g dry matter) and two KF of their allocated KF intervention (25.6 g dry matter of green KF and 27.2 g dry matter of gold KF). The pigs were anesthetized through intramuscular injection in the neck at a dose rate of 120 μL/kg of an anesthetic cocktail (Zoletil 100 (50 mg/mL), ketamine (50 mg/mL), and xylazine (50 mg/mL)). Pigs were transferred from the cage to the surgery room, where blood samples (~4 mL) from the anterior jugular vein (which carries blood from the brain to the heart) were collected. Blood samples were collected into ethylenediaminetetraacetic acid (EDTA)-containing tubes (BD Vacutainer^®^, Becton, Franklin Lakes, NJ, USA) and kept on ice for 10 min before centrifugation for separation of plasma at 3000× *g* for 5 min. The plasma samples were frozen on dry ice immediately and stored at −80 °C until analysis. Once blood samples were collected, the pigs were euthanized by intracardial injection of sodium pentobarbitone (0.3 mL/kg BW of Pentobarb 300; Provet NZ Pty Ltd., Christchurch, New Zealand) [23].

Once pigs were euthanized, a registered veterinarian removed the head from the body and placed it into clamps. The skull plate was cut carefully using a bone saw, ensuring the brain remained undamaged. Once the skull plate was cut, the plate was levered up to give access to the brain. The brain was carefully scooped out, and the two lobes were gently pulled apart. The left hemisphere was used for consistency with other studies [25], and the brain stem (regulation of vital life functions, e.g., breathing, blood pressure), corpus striatum (reward and reinforcement circuit), hippocampus (role in learning and memory), and prefrontal cortex (central role in cognitive control functions) were carefully dissected out. All the samples were snap-frozen in liquid nitrogen, kept on dry ice, and stored at −80 °C for analysis. All brain samples were collected within 15 min of euthanasia.

### 2.3. Biochemcial Measures

Prior to biochemical analyses, the brain tissue regions were lysed in Neuronal Protein Extraction Reagent (N-PER™, Thermo Scientific, Waltham, MA, USA) with a cocktail of protease and phosphatase inhibitors included (Pierce Protease and Phosphatase Inhibitor Mini Tablets, Thermo Scientific, Waltham, MA, USA) at a ratio of 10 mL per 1 g of tissue. This mix was then placed on ice for 10 min and centrifuged at 10,000× *g* for 10 min at 4 °C. The Bradford assay (Bio-Rad Laboratories, Hercules, CA, USA) measured the supernatant protein concentrations with bovine serum albumin as the standard. As described in the following sections, the supernatant was aliquoted and stored at −80 °C for downstream analyses.

#### 2.3.1. Plasma Oxidative Stress Measures

Plasma MDA levels (lipid peroxidation biomarker) and plasma protein carbonyls (protein peroxidation biomarker) were used to evaluate the oxidative stress state of animals. MDA was assessed by high-performance liquid chromatography (HPLC) using a modified method described by Karatepe [26] against MDA standards. MDA levels were presented as μmol/L. Protein carbonyls were measured using the modified version of a colorimetric end-point assay described by Levine et al. [27]. Carbonyl levels were calculated as nmol/L.

#### 2.3.2. Plasma and Brain Tissue Antioxidant Measures

##### Oxidative Potential Assay (OPA)

ROS-generating potential capacity was assessed using a modified carboxy-dihydro-2′,7′-dichlorohydrofluorescein diacetate (carboxy-H2DCFDA) kinetic assay described by Wang and Joseph [28]. The assay consisted of hydrolyzing 10 μM carboxy-H_2_DCFDA (Thermo Scientific, Waltham, MA, USA) into the product dichlorofluorescein (DCF), which is fluorescent when oxidized. Carboxy-H2DCFDA was hydrolyzed with an equal volume of methanol and 1 M potassium hydroxide for 1 h at room temperature. An amount of 5 μL of diluted plasma (1:5) and brain lysates (1:100) was added to black 96-well plates, and then 1 μM H_2_O_2_ was added. Immediately after, 5 μL of DCF was added to each well. The changes in fluorescence intensity were measured over 5 min at 37 °C using a fluorescence plate reader with excitation and emission wavelengths of 485 and 528 nm, respectively. All plasma and brain extracts were assayed in triplicate, with the coefficient of variation (CV) of replicate measures being <10%. Data were presented as the percentage change in relative fluorescence intensity after 5 min (%ΔFI_5min_) and for brain tissue standardized to a milligram protein content.

##### Ferric-Reducing Antioxidant Power (FRAP)

The FRAP assay used the standard method described by Benzie and Strain [29]. Briefly, plasma and brain lysates diluted in acetate buffer (1:8) were added to an equal amount of FRAP reagent (containing TPTZ [2,4,6-tripyridyl-s-triazine] and ferric chloride in a hydrochloric acid solution). After 15 min of incubation at room temperature, the absorbance was measured on a plate reader set at a wavelength of 593 nm. All plasma and brain extracts were assayed in triplicate, with the CV of replicate measures being <10%. Plasma and brain lysate antioxidant capacities were measured against a standard curve of Trolox (Merck, Auckland, New Zealand), calculated as μM Trolox equivalents and standardized to a milligram protein content of plasma and brain tissue.

##### Oxygen Radical Absorbance Capacity (ORAC)

The ORAC assay used the standard method described by Prior et al. [30]. Briefly, plasma and brain lysates were diluted in PBS (plasma 1:40 and brain 1:40), and fluorescein was added and incubated at 37 °C for 30 min. 2,2′-Azobis(2-amidinopropane) dihydrochloride (AAPH) was added, and the fluorescence intensity was measured on a plate reader with excitation and emission wavelengths of 485 and 520 nm, respectively, for 90–120 min at 2 min intervals. All plasma and brain extracts were assayed in triplicate, with the CV of replicate measures being <10%. The ORAC was measured against the standard curve of Trolox (Merck, Auckland, New Zealand), calculated as μM Trolox.

##### Vitamin C (Ascorbate) Concentration

The ascorbate content of plasma and brain tissue was measured on reverse-phase HPLC with a Synergi 4 micron Hydro-RP 80-A column and an ESA coulochem II electrochemical detector [31]. Ascorbate concentrations in plasma were expressed in μM, and those in brain tissue were expressed in nmol/mg of wet-weight tissue.

##### Catalase Activity

Catalase activity was determined in the brain regions using the Catalase Colorimetric Activity Kit (Invitrogen™, Waltham, MA, USA). A bovine catalase standard was used to generate a standard curve for the assay and all tissue lysates read off the standard curve. Brain lysates were diluted at 1:20 in the provided assay buffer and added to the wells of a half-area transparent plate. H_2_O_2_ was added to each well, and the plate was incubated at room temperature for 30 min. Following this incubation, the supplied colorimetric detection reagent was added, followed by diluted horseradish peroxidase and incubation at room temperature for 15 min. The colored product was read at 560 nm. All brain extracts were assayed in triplicate, with the CV of replicate measures being <10%. The activity of catalase was standardized to protein and expressed as U/mg protein.

##### Superoxide Dismutase Activity

SOD activity was determined in the brain regions using a SOD Activity Assay Kit (Sigma-Aldrich, St. Louis, MO, USA), based on the procedure described by Peskin and Winterbourn [32]. A bovine SOD standard was used to generate a standard curve for the assay and all tissue lysates read off the standard curve. Briefly, samples were diluted at 1:1000 with the provided assay buffer and added to the wells. The activity of SOD was determined by measuring the decrease in superoxide anions (generated by the enzyme xanthine oxidase [XO]). The superoxide anions react with water-soluble tetrazolium (WST) dye, producing a color read at a 450 nm wavelength. The decrease in signal is proportional to SOD inhibition activity. All brain extracts were assayed in triplicate with the CV of replicate measures being <10%. The activity of SOD was standardized to protein and expressed as U/mg protein.

##### Western Blot Analysis of Antioxidant Enzymes

A Western blot analysis was conducted to examine the protein levels of antioxidants in the brain regions. The biochemical analysis section described the brain lysates used for this analysis. Aliquots containing 25 μg of total protein were boiled in a loading buffer containing 150 mM Tris (pH 6.8), 3 mM DTT, 6% SDS, 0.3% bromophenol blue, and 30% glycerol. Catalase was resolved into wells of 12% Mini-PROTEAN^®^ TGX™ Precast Protein Gels (Bio-Rad Laboratories, Hercules, CA, USA). SOD enzymes were resolved into wells of 4–20% Mini-PROTEAN^®^ TGX™ Precast Protein Gels (Bio-Rad Laboratories, Hercules, CA, USA). Electrophoresis of all gels was conducted in Bio-Rad Criterion cell systems at room temperature under normal running buffer (Tris/Glycine/SDS buffer).

Proteins from the gels were blotted onto Immun-Blot^®^ PVDF Membrane (Bio-Rad Laboratories, Hercules, CA, USA) and then blocked with 5% nonfat dry milk powder in PBS-Tween (0.08 M Na_2_PO_4_, 0.02 M NaH_2_PO_4_, 0.1 M NaCl, 0.1% Tween 20). After washing with PBS-Tween three times for 10 min each, the membranes were cut at approximately 50 kD and 30 kD and incubated with primary polyclonal antibodies for 1 h at room temperature. Polyclonal antibodies used included rabbit anti-catalase (Thermo Scientific, Waltham, MA, USA, BS-2302R), rabbit anti-SOD2 (Thermo Scientific, Waltham, MA, USA, PA1-31072), rabbit anti-SOD1 (Abcam, Cambridge, UK, ab13498), and rabbit anti-GAPDH (Thermo Scientific, Waltham, MA, USA, PA5-85074) diluted to 1:1000, 1:5000, 1:5000, and 1:10,000, respectively, in PBS-Tween. After washing with PBS-Tween, membranes were incubated with a secondary antibody, mouse anti-rabbit IgG HRP conjugate (BioLegend, San Diego, CA, USA, Cat. 410404), diluted to 1:2000 in PBS-Tween, for 1 h at room temperature. The secondary antibody bound to the membrane was detected with an ECL Western blot substrate kit (Bio-Rad Laboratories, Hercules, CA, USA). The resulting luminescent bands were captured with a G:Box Chemi HR16 (Syngene, Cambridge, UK). Relative quantitation was calculated by normalization to the housekeeping gene GAPDH.

##### AChE Activity

AChE activity was determined using an Amplex™ Acetylcholine/Acetylcholinesterase Assay Kit (Thermo Scientific, Waltham, MA, USA) according to the manufacturer’s instructions. Briefly, Amplex Red substrate was added to tissue lysates and H_2_O_2_ standards (0.01–2 mM) and phosphate buffer control. The change in fluorescence was measured at 37 °C over 10 min (530–560 and 590 nm excitation and emission wavelengths, respectively) on a fluorescence plate reader. Brain regional AChE activity was calculated against H_2_O_2_ standards and expressed as nM H_2_O_2_ produced/μg protein/min. All brain extracts were assayed for AChE activity in triplicate, with the CV of replicate measures being <10%.

### 2.4. Data Analysis

Statistical analyses were performed using SPSS software (version 28; IBM, Armonk, NY, USA). Effects of treatment on plasma biomarkers were determined using a one-way ANOVA. Outliers were assessed by examination of studentized residuals for values greater than ±3. Normality was assessed by Shapiro–Wilk’s test (*p* > 0.05). The effects of treatment and brain region and their interaction were determined using a repeated-measures mixed-effects model. Residual plots were inspected to confirm that the normality and constant variance model assumptions were met. An unstructured covariance structure was used to account for the repeated regions. Post hoc comparisons, adjusted for multiple comparisons using Bonferroni’s correction, were performed when there were significant main or interaction effects. For some antioxidant measures, data were log-transformed before analysis to stabilize the variance. Statistical significance was accepted at a probability inferior to 0.05 (*p* < 0.05). A statistical trend was observed with a *p*-value between 0.05 and 0.10. All data are presented as means ± standard error of means (SEM). Pearson correlation was also performed between all regional brain biomarkers and nutrient intakes. Furthermore, where interaction effects were present in brain regions, Pearson correlation was also performed with corresponding plasma measures. Correlations with *p* < 0.05 and R-value > ±0.5 were considered [33].

## 3. Results

### 3.1. Plasma Oxidative and Antioxidant Markers

**Figure 1:**
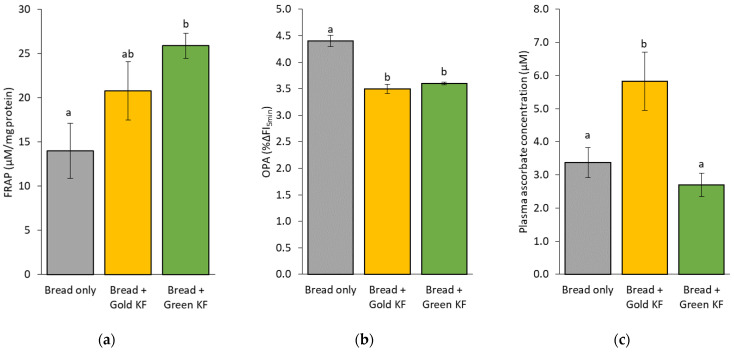
Estimated marginal means and standard error of means (SEM) for plasma ferric-reducing antioxidant potential (FRAP) values (**a**), oxidative potential assay (OPA) (**b**), and ascorbate concentrations (**c**) in a growing pig model in response to the bread, bread with gold kiwifruit (KF), and bread with green KF treatments. Bars in each graph without a common letter are significantly different (*p* < 0.05).

Treatment effects were found for plasma FRAP [*F*(2, 14) = 4.272, *p* = 0.0036], OPA [*F*(2, 14) = 33.214, *p* < 0.0005], and plasma ascorbate concentrations [*F*(2, 15) = 7.402, *p* = 0.006]. FRAP was higher in pigs fed bread + green KF (25.9 ± 1.4 μM) compared to pigs fed bread only (14.0 ± 3.1 μM; post hoc *p* = 0.030) (Figure 1a). OPA was higher after bread only (4.4 ± 0.11 ΔFI_5min_) compared to both bread + gold KF (3.5 ± 0.09 ΔFI_5min_; post hoc *p* < 0.005) and bread + green KF (3.6 ± 0.03 ΔFI_5min_; post hoc *p* < 0.005) (Figure 1b). Ascorbate concentrations were higher in pigs fed bread + gold KF (5.8 ± 0.9 μM) compared to pigs fed with bread + green KF (2.7 ± 0.4 μM; post hoc *p* = 0.006) and bread only (3.4 ± 0.5 μM; post hoc *p* = 0.030) (Figure 1c). There were no effects of treatment on plasma oxidative stress markers (MDA and protein carbonyls) (Table S1).

### 3.2. Antioxidant Enzyme Activity and Capacity in Different Brain Regions

A summary of antioxidant and enzyme activity measures is presented in Table 2. The main effects of the brain region were identified for ORAC [*F*(3, 15) = 27.03, *p* < 0.001], OPA [*F*(3, 15) = 41.07, *p* < 0.001], catalase activity [*F*(3, 15) = 30.54, *p* < 0.001], SOD activity [*F*(3, 15) = 211.87, *p* < 0.001], SOD2 expression [*F*(3, 15) = 6.24, *p* = 0.006], ascorbate concentration [*F*(3, 15) = 379.62, *p* < 0.001], and AChE activity [*F*(3, 15) = 3045.85, *p* < 0.001].

Irrespective of treatment, ORAC was significantly higher in the brain stem (mean ± SEM across brain regions, 39.13 ± 1.70 µM Trolox/mg protein) compared to corpus striatum (23.70 ± 1.26 µM Trolox/mg protein, post hoc *p* < 0.005), hippocampus (27.86 ± 1.70 µM Trolox/mg protein; post hoc *p* < 0.005), and prefrontal cortex (26.39 ± 1.49 µM Trolox/mg protein; post hoc *p* < 0.005). OPA values were higher in the prefrontal cortex (mean ± SEM across brain regions, 29.49 ± 5.18 ΔFI_5min_) than in the hippocampus (8.97 ± 1.81 ΔFI_5min_), corpus striatum (4.93 ± 2.15 ΔFI_5min_), and brain stem (−1.42 ± 1.30 ΔFI_5min_).

Catalase activity was significantly higher in the brain stem (mean ± SEM across brain regions, 16.01 ± 0.42 U/mg protein) compared with all other regions (corpus striatum, 12.16 ± 0.22 U/mg protein; hippocampus, 12.67 ± 0.43 U/mg protein; prefrontal cortex, 12.91 ± 0.45 U/mg protein). SOD activity was significantly lower in the brain stem (101.72 ± 10.57 U/mg protein) compared with the corpus striatum (351.45 ± 21.85 U/mg protein), hippocampus (665.03 ± 23.99 U/mg protein), and prefrontal cortex (477.39 ± 51.41 U/mg protein). SOD2 protein expression was higher in the corpus striatum (1.26 ± 0.09 relative expression) than in the hippocampus (0.92 ± 0.06 relative expression). Ascorbate concentration was significantly higher in the hippocampus (1.28 ± 0.02 nmol/mg tissue) compared with the prefrontal cortex (1.02 ± 0.04 nmol/mg tissue), corpus striatum (0.79 ± 0.03 nmol/mg tissue), and brain stem (0.65 ± 0.01 nmol/mg tissue). AChE activity was significantly higher in the corpus striatum (93.30 ± 0.51 nM H_2_O_2_/µg protein/min) compared with the prefrontal cortex (27.10 ± 0.64 nM H_2_O_2_/µg protein/min), hippocampus (38.84 ± 0.46 nM H_2_O_2_/µg protein/min), and brain stem (46.09 ± 0.65 nM H_2_O_2_/µg protein/min).

### 3.3. Antioxidant Enzyme Activity and Capacity after Kiwifruit Intake

**Figure 2:**
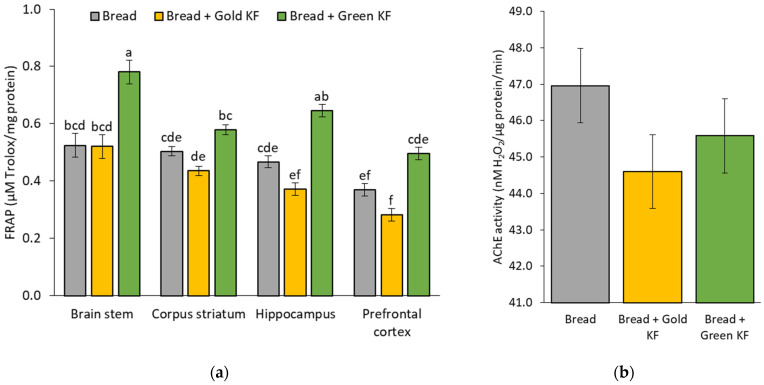
Estimated marginal means and standard error of means (SEM) for brain regional ferric-reducing antioxidant potential (FRAP) values (**a**) and acetylcholinesterase (AChE) activity across all brain regions (**b**) in a growing pig model in response to bread, bread with gold kiwifruit (KF), and bread with green KF treatments. Values in the graphs that share the same letters in each region are not significantly different (*p* < 0.05).

An interaction effect of treatment by brain region was found for FRAP [F(6, 15) = 4.33, *p* = 0.010]. Within the brain stem and prefrontal cortex, FRAP was significantly higher after bread + green KF compared to both bread + gold KF and bread only (*p* < 0.05; Figure 2a). Furthermore, the greatest FRAP was recorded in the brain stem when bread + green KF was consumed, and the lowest FRAP was in the prefrontal cortex when bread + gold KF was consumed.

The analysis also determined a trend towards a main effect of treatment for AChE activity [F(2, 15) = 2.90 *p* = 0.086; Figure 2b]. AChE activity tended to be lower in the gold KF treatment group (44.60 ± 1.02 nM H_2_O_2_/µg protein/min) compared to bread only (46.96 ± 1.02 nM H_2_O_2_/µg protein/min, *p* = 0.090). No additional interaction or treatment effects were observed on any other measures of antioxidants in the brain regions.

### 3.4. Correlations between Plasma and Brain Regional FRAP

**Figure 3:**
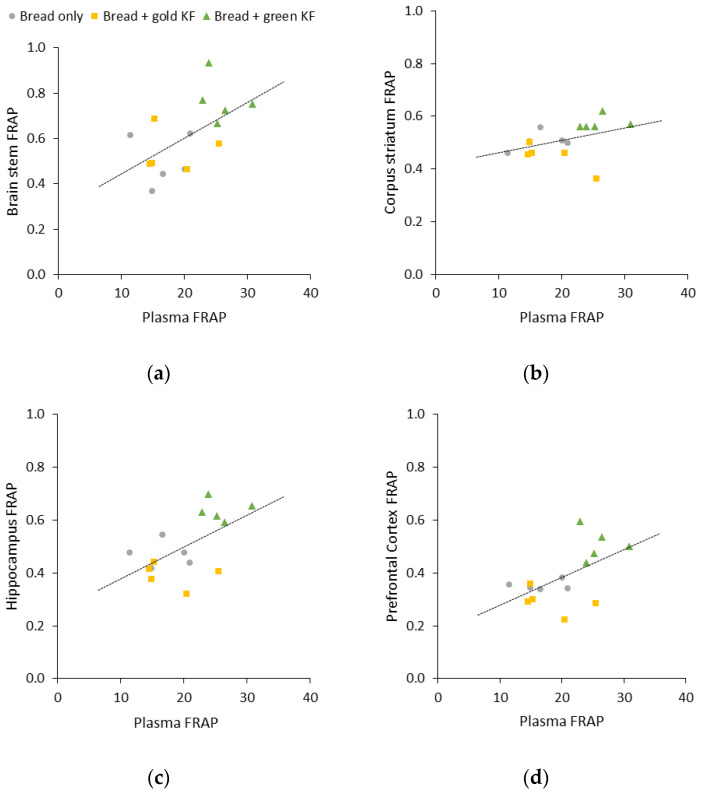
Linear correlations between plasma ferric-reducing antioxidant potential (FRAP) and brain regional FRAP in the brain stem (**a**) (r = 0.57, *p* = 0.027), corpus striatum (**b**) (r = 0.41, *p* = 0.135), hippocampus (**c**) (r = 0.59, *p* = 0.022), and prefrontal cortex (**d**) (r = 0.57, *p* = 0.028) in a growing pig model (*n* = 15) fed a bread-based diet alone or supplemented with gold or green kiwifruit (KF). Single outliers per treatment group were detected with scatter plots, leaving each treatment group with an *n* = 5.

With treatments and interaction having a significant effect on FRAP measurements, a correlation analysis between plasma FRAP and brain regional FRAP was conducted. There was a statistically significant positive correlation between plasma FRAP and brain stem FRAP (r = 0.57, *p* = 0.027; Figure 3a), hippocampus FRAP (r = 0.59, *p* = 0.022; Figure 3c), and prefrontal cortex FRAP (r = 0.57, *p* = 0.028; Figure 3d). However, there was no statistically significant relation between plasma FRAP and corpus striatum FRAP (r = 0.41, *p* = 0.135; Figure 3b).

### 3.5. Correlation between Nutrient Intake and Antioxidant Capcity in the Brain Regions

**Figure 4:**
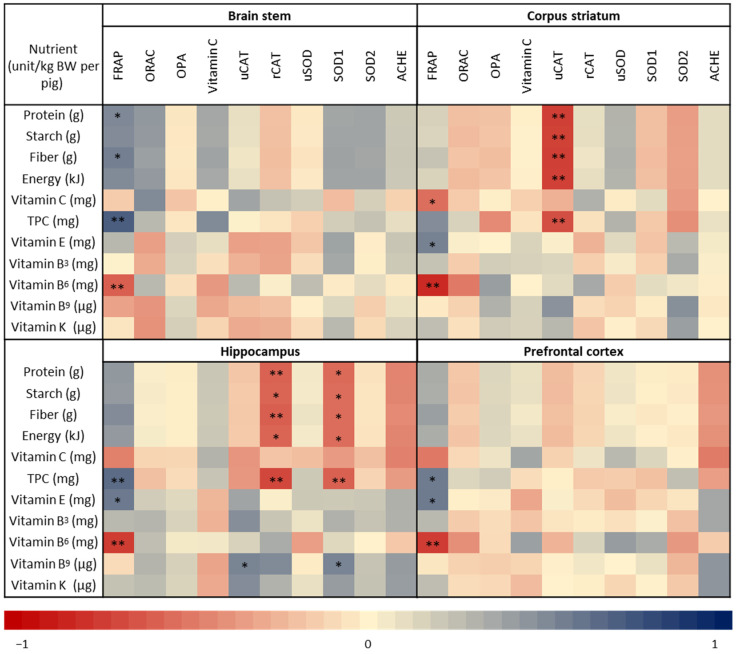
Pearson correlation plot between nutrient intake and biochemical markers in different brain regions (brain stem, corpus striatum, hippocampus, and prefrontal cortex) obtained from pigs fed bread only, bread with gold kiwifruit (KF), or bread with green KF. The plot is color-coded to indicate the strength and direction of the correlation, with blue representing a positive correlation and red representing a negative correlation. Asterisk (*) symbols indicate different levels of statistical significance. * *p* < 0.05; ** *p* < 0.01. TPC: total polyphenol content, FRAP: ferric-reducing antioxidant potential, ORAC: oxygen radical absorbance capacity, OPA: oxidative potential assay, uCAT: catalase activity, rCAT: catalase expression, uSOD: superoxide dismutase activity, AChE: acetylcholinesterase activity.

Pearson correlation analysis revealed significant correlations between FRAP and various macro- and micronutrients in all brain regions (Figure 4). FRAP in the corpus striatum, hippocampus, and prefrontal cortex was significantly positively correlated with vitamin E intake (r = 0.54, 0.58, and 0.57, respectively). Similarly, FRAP in the brain stem, hippocampus, and prefrontal cortex significantly correlated with total polyphenol content (TPC) intake (r = 0.71, 0.57, and 0.57, respectively). FRAP was also significantly correlated with protein and fiber intake in the brain stem (r = 0.51 and 0.55, respectively). Conversely, FRAP in all the brain regions was negatively correlated with vitamin B6 intake: brain stem (r = −0.60), corpus striatum (r = −0.83), hippocampus (r = −0.75), and prefrontal cortex (r = −0.75).

Protein, starch, fiber, energy, and TPC intakes were found to be significantly negatively correlated with catalase activity in the corpus striatum (r = −0.74, −0.73, −0.74, −0.73, and −0.67, respectively) and catalase protein expression in the hippocampus (r = −0.59, −0.58, −0.60, −0.58, and −0.70, respectively). SOD1 protein expression in the hippocampus also significantly negatively correlated with protein, starch, fiber, energy, and TPC intakes in the hippocampus (r = −0.56, −0.55, −0.55, −0.55, and −0.60, respectively). Conversely, catalase activity and SOD1 protein expression in the hippocampus were significantly correlated with vitamin B9 intake (r = 0.52 and 0.53, respectively).

## 4. Discussion

This study sought to investigate how the addition of green or gold KF to a bread-based diet affected the antioxidant capacity of different brain regions in pigs. The current study shows that KF supplemented to pigs consuming a bread-based diet resulted in changes in antioxidant measures in plasma and brain regions. Green KF increased antioxidant capacity and decreased oxidative-generating potential in plasma, while gold KF increased plasma ascorbate concentrations and decreased oxidative-generating potential in plasma. Additionally, green KF increased chemical antioxidant capacity in all brain regions, and gold KF showed a trend towards reducing AChE activity. Findings from this study also highlight the differences in antioxidant capacity, antioxidant enzyme activity, and protein expression between brain regions. This finding is supported by higher catalase in the prefrontal cortex and SOD activities in the brain stem compared with other brain regions. Conversely, oxidative generating potential and AChE activity were lower in the brain stem and prefrontal cortex, respectively, compared with other brain regions. Interestingly, FRAP was found to be positively correlated with total polyphenol intake in all brain regions. Altogether, these findings suggest the effect of green KF consumption in improving brain antioxidant capacity may have benefits for neuroprotection from oxidative stress.

One of the promoted health benefits of KF is its potential as a dietary antioxidant. This benefit may be attributed to the concentrations of antioxidant micronutrients in KF (i.e., vitamin C, vitamin E, carotenoids, and polyphenolics) that are chemically powerful antioxidants. KF has also been reported to activate the nuclear factor erythroid 2-related factor (Nrf2) signaling pathway [34] responsible for inducing the transcription of antioxidant enzymes and reducing transcription factor nuclear factor kappa B (NF-κB) [35], a key transcription factor that regulates the inflammatory response in cells.

### 4.1. Effects of Kiwifruit on Peripheral Antioxidant Capacity

The increased plasma antioxidant potential after the KF treatments reported from the current study are consistent with KF interventions in humans, which showed an increase in plasma FRAP [13] and ORAC [12]. The increase in FRAP observed in this study has also been observed in pigs fed a wheat diet with polyphenol-rich fruit (blackcurrants) [36]. The interpretation of these results depends on the method of antioxidant measure. Both methods measure a different ‘antioxidant capacity’, which may explain the divergent results (significant effect on FRAP but not on ORAC). A FRAP measurement indicates the reducing capacity (electron transfer), while an ORAC measurement indicates the capacity to scavenge radicals (hydrogen atom transfer) [37]. The results suggest that KF might impact electron transfer, which could lead to a decrease in the generation of ROS. This observation is consistent with the finding that KF treatments reduced the ROS generating potential capacity and may prime the body to respond to periods of oxidative stress and protect from the detrimental effects of excessive ROS. This interpretation is supported by a recent study reporting reduced levels of oxidative stress in women who consumed a gold KF drink before engaging in an acute high-intensity exercise [38].

### 4.2. Effects of Kiwifruit on Brain Antioxidant Capacity

Regulating oxidative stress in the brain is crucial for maintaining cognitive health, as excessive oxidative damage has been implicated in neurodegenerative disorders. Research has shown that dietary interventions, such as increased intake of fruits and vegetables, can enhance antioxidant capacity, as evidenced by improvements in FRAP [39], potentially mitigating neurodegenerative risks. The hippocampus and prefrontal cortex are reported to be the most susceptible brain regions to undergo functional decline following exposure to oxidative stress [40]. The susceptibility of these brain regions to oxidative stress has been linked to behavioral and cognitive deficits in rodents exposed to stress [41].

While detrimental effects of oxidative stress on these brain regions and their cognitive consequences have been characterized, it is evident that these regions may also be most reactive to oxidative stress. OPA results showed that both the prefrontal cortex and the hippocampus showed the highest ROS generating capacity of the four regions tested. Like previous studies in rats [42], SOD activity was highest in the hippocampus of pigs compared with other brain regions, indicating that the higher endogenous antioxidant activity in the hippocampus provides some resilience to oxidative stress.

KF consumption has been demonstrated to have neuroprotective effects in in vitro models and in rodents, attenuating cellular ROS and protecting cells from the detrimental effects of oxidative stress [14,15,16,17]. The reduction in oxidative stress and increase in antioxidant markers from KF consumption corresponded with reduced decrements in cognitive behavioral assessments in stressed rodents [14,15]. In this study, green KF supplementation induced greater antioxidant protective potential in all brain regions than gold KF and bread only. Besides having differing vitamin C, vitamin E, and polyphenol contents, as mentioned previously, actinidin is required for the cleavage of kiwellin, a minor protein in KF, into KiTH and kissper [19]. The latter has demonstrated anti-inflammatory properties [21], potentially leading to the downregulation of systemic ROS. Whether these proteins are responsible for the beneficial effects in the brain has yet to be determined and could be explored in future research.

Like findings from human dietary intervention studies [9,11,13,31,43,44,45,46,47], the current findings showed that consuming gold KF increased plasma concentrations of ascorbate in pigs. However, this result was not observed in brain tissue where KF consumption had no effect on regional brain ascorbate concentrations. This could be because pigs, in contrast to humans, can synthesize ascorbate, and so it is not considered essential for the species [48]. Additionally, the brain retains higher concentrations of ascorbate than does plasma, and these brain stores are maintained throughout states of deficiency [48]; thus, any additional dietary intake of ascorbate would result in no observable changes in the brain.

Mapping ascorbate distribution in the human brain [49,50], like the results observed in this study, showed the highest concentrations of ascorbate in the hippocampus, followed by the prefrontal cortex. The transportation of ascorbate can explain the differential concentrations of ascorbate across the brain. Ascorbate is transported by the sodium-dependent vitamin C transporter type 2 (SVCT2) [51], which is more abundant in brain regions that contain many neurons, such as the cortex and hippocampus [52].

Compared to rodents [42], the present findings showed that AChE activity was the highest in the corpus striatum region in pigs. This is because, in this region, cholinergic interneurons spontaneously release ACh and AChE [53]. Rodent studies reveal that curcumin [54] and green tea [55] can inhibit AChE activity, hinting at their cognitive health benefits. KF extracts inhibit AChE in vitro [56]. A trend in reduced AChE activity across the whole brain after gold KF treatments was observed in the current results, corresponding with the findings in the brains of high-fat-diet-induced obese mice supplemented with *A. arguta* extracts [14]. This finding could be due to the polyphenols that gold KF provides (epicatechin), which are present in lower concentrations in green KF [57]. These polyphenols have been shown to have inhibitory effects on AChE [58,59] and warrant further exploration.

### 4.3. Correlations between Peripheral and Brain Antioxidant Capacity

The strong correlations between FRAP in plasma and in the different brain regions suggest that plasma FRAP measurement may serve as a potential biomarker of brain antioxidant capacity. Whether increased peripheral FRAP directly influences the antioxidant capacity of brain regions is unclear, and further research is needed to elucidate the clinical relevance of this correlation.

### 4.4. Assocations between Kiwifruit Composition and Total Antioxidant Capacity

The analysis revealed that increases in antioxidant potential in the brain regions were strongly correlated with higher concentrations of total polyphenols and vitamin E. Given that both vitamin E and polyphenols are known to influence FRAP [60] and that both these constituents are present at higher concentrations in green KF than in gold KF (Table 1), it is likely that the greater concentrations of these constituents consumed by pigs explains the greater FRAP in brain tissue. This was observed in pigs that consumed green KF compared with those who consumed gold KF and bread-only diets.

### 4.5. Strengths, Limitations, and Future Studies

This study was the first to assess the effect of KF administered with a bread-based diet on the endogenous antioxidant systems of plasma and brain regions in a pig model. Although the results do not demonstrate significant impacts on the antioxidant systems, they provide valuable evidence, which could direct further research. A key strength of this study is the use of pigs; besides having digestive physiology similar to humans [61], the pig brain is also more anatomically similar to humans than are mice or rat models. Like humans, pigs have gyrencephalic brains, and their white-to-gray matter ratio is 60:40 [62]. Moreover, pig and human brains have homologous resting-state networks [63]. Additionally, a notable strength of this study lies in the fact that the dosage of fresh KF used mirrors what is commonly considered a standard serving in humans, enhancing the translational relevance of the results compared to in vitro experiments or studies involving rodent models using extracts.

Nevertheless, this study has several limitations. Firstly, caution is advised when interpreting the results as the research was conducted on healthy pigs, and it is unclear whether KF provides any additional benefits in diseased or unhealthy individuals. Secondly, while previous studies in rodents showed enhanced antioxidant systems, the models used in these studies had increased oxidative stress due to external stressors like a high-fat diet [14], lead exposure [15], and causative agent Aβ injection [16], which may have amplified the protective effects of KF on antioxidant systems. Thirdly, this study’s sample size was constrained due to its amalgamation with another study. Future research should incorporate a larger animal cohort to enhance result reliability. Our findings suggest the efficacy of green KF in elevating plasma antioxidant protection. However, to validate these results, a sample size of 40 (20 per KF treatment and control, power = 80%, α = 0.05) is required based on plasma FRAP mean and standard deviation comparisons between green KF with bread and bread alone. Additionally, the diet used in this study was limited to bread only, which is not representative of a normal diet for humans.

As previously highlighted, future studies on the neuroprotective effects of KF in pig models should consider manipulating stress through dietary interventions (Western or high-fat) [64,65] or chronic stressors [66]. Cognitive measures [67] and the investigation of specific components of KF, such as pure vitamins or KF skin, similar to other studies [68], could provide valuable insights on which KF constituents contribute to the antioxidant modulatory properties of KF in the brain. Comparisons with other polyphenol-rich fruits or purified polyphenol compounds could also be informative. Assessing additional biomarkers like brain-derived neurotrophic factor (BDNF), which plays a crucial role in neuronal growth and survival, would further enhance our understanding [69]. Human clinical studies, particularly with populations experiencing high levels of stress, could provide relevant information. Administering KF or a placebo prior to cognitive tests and assessing antioxidant status through blood samples would be a valuable approach, consistent with similar studies [70].

## 5. Conclusions

Oxidative stress caused by the inability of endogenous antioxidant systems to counteract ROS formation in the brain leads to neurodegeneration, resulting in cognitive and behavioral decline. Overall, this study is the first to demonstrate the efficacy of green and gold KF consumption in differentially augmenting the antioxidant capacity of plasma and brain regions in growing pigs. Specifically, consuming green KF increased FRAP and gold KF tended to reduce AChE activity across all brain regions. These findings would suggest the potential neuroprotective effect of green or gold KF during oxidative stress by augmenting the brain’s antioxidant capacity. Further studies would need to be conducted to elucidate the neuroprotective potential of KF during stress.

## Figures and Tables

**Table 1:** Average determined nutrient (g/kg of body weight (BW) per pig) intake of diets with bread only, bread + gold kiwifruit (KF), and bread + green KF over the supplementation period.

Composition (Unit/kg BW per Pig)	Bread Only	Bread + Gold KF	Bread + Green KF
Dry matter (g)	40.81	40.61	41.68
Protein (g)	2.80	2.91	3.00
Starch (g)	14.34	14.82	15.23
Fiber (g)	2.09	2.15	2.24
Gross energy (kJ)	493.91	510.51	525.12
Vitamin C (mg)	0.27	13.27	4.69
Vitamin E (mg)	3.38	2.87	3.30
Vitamin K (μg)	0.18	0.15	0.17
Vitamin B3 (mg)	1.64	1.48	1.56
Vitamin B6 (mg)	0.30	0.31	0.28
Vitamin B9 (μg)	23.06	19.16	19.44
Total polyphenol content (mg GAE)	0.22	0.63	1.17
GAE: gallic acid equivalents			

**Table 2:** Measures of antioxidant and enzyme activities across brain regions in a growing pig model in response to the intake of bread, bread with gold kiwifruit (KF), and bread with green KF treatments.

Measure	Treatment	Brain Stem	Corpus Striatum	Hippocampus	Prefrontal Cortex	Factors	*F*	*p*
FRAP (µM/mg protein)	Bread only	0.52 ± 0.04 ^bcd^	0.50 ± 0.02 ^cde^	0.47 ± 0.02 ^cde^	0.37 ± 0.02 ^ef^	Treatment	52.52	<0.001
	Bread + gold KF	0.52 ± 0.04 ^bcd^	0.44 ± 0.02 ^de^	0.37 ± 0.02 ^ef^	0.28 ± 0.02 ^f^	Brain region	49.48	<0.001
	Bread + green KF	0.78 ± 0.04 ^a^	0.58 ± 0.02 ^bc^	0.65 ± 0.02 ^ab^	0.50 ± 0.02 ^cde^	Treatment × regions	4.33	0.010
ORAC (µM/mg protein)	Bread only	34.01 ± 2.94	23.40 ± 2.18	29.86 ± 2.94	26.01 ± 2.57	Treatment	0.27	0.764
	Bread + gold KF	43.64 ± 2.94	22.21 ± 2.18	27.37 ± 2.94	25.20 ± 2.57	Brain region	27.03	<0.001
	Bread + green KF	39.73 ± 2.94	25.50 ± 2.18	26.34 ± 2.94	27.98 ± 2.57	Treatment × regions	1.72	0.184
OPA (%ΔFI_5min_/mg protein)	Bread only	−0.47 ± 2.26	8.76 ± 3.72	10.19 ± 3.13	24.49 ± 8.97	Treatment	0.01	0.995
	Bread + gold KF	−2.65 ± 2.26	6.76 ± 3.72	8.13 ± 3.13	29.72 ± 8.97	Brain region	41.07	<0.001
	Bread + green KF	−1.14 ± 2.26	−0.72 ± 3.72	8.59 ± 3.13	34.26 ± 8.97	Treatment × regions	0.73	0.631
Ascorbate (nmol/mg tissue)	Bread only	0.64 ± 0.02	0.80 ± 0.04	1.25 ± 0.03	1.00 ± 0.07	Treatment	1.00	0.392
	Bread + gold KF	0.65 ± 0.02	0.77 ± 0.04	1.31 ± 0.03	1.12 ± 0.07	Brain region	379.62	<0.001
	Bread + green KF	0.69 ± 0.02	0.79 ± 0.04	1.30 ± 0.03	0.97 ± 0.07	Treatment × regions	1.25	0.338
Catalase activity (U/mg protein)	Bread only	15.53 ± 0.73	13.08 ± 0.37	13.92 ± 0.75	13.07 ± 0.78	Treatment	1.11	0.354
	Bread + gold KF	17.04 ± 0.73	12.10 ± 0.37	11.98 ± 0.75	12.62 ± 0.78	Brain region	30.54	<0.001
	Bread + green KF	15.72 ± 0.73	11.30 ± 0.37	12.10 ± 0.75	13.04 ± 0.78	Treatment × regions	2.10	0.114
Catalase (relative expression)	Bread only	1.02 ± 0.07	0.98 ± 0.11	1.11 ± 0.06	1.01 ± 0.09	Treatment	2.53	0.113
	Bread + gold KF	1.11 ± 0.07	1.17 ± 0.11	0.97 ± 0.06	1.02 ± 0.09	Brain region	0.78	0.525
	Bread + green KF	1.01 ± 0.07	0.95 ± 0.11	0.79 ± 0.06	0.93 ± 0.09	Treatment × regions	1.26	0.331
SOD activity (U/mg protein)	Bread only	101.5 ± 18.3	327.1 ± 37.9	664.2 ± 41.6	458.8 ± 89.1	Treatment	0.30	0.746
	Bread + gold KF	115.4 ± 18.3	334.6 ± 37.9	623.6 ± 41.6	578.9 ± 89.1	Brain region	211.87	<0.001
	Bread + green KF	88.3 ± 18.3	392.7 ± 37.9	707.3 ± 41.6	394.5 ± 89.1	Treatment × regions	0.92	0.510
SOD1 (relative expression)	Bread only	1.10 ± 0.30	0.96 ± 0.10	1.21 ± 0.07	1.04 ± 0.21	Treatment	0.41	0.668
	Bread + gold KF	0.80 ± 0.30	1.01 ± 0.10	0.97 ± 0.07	1.15 ± 0.21	Brain region	0.53	0.669
	Bread + green KF	1.34 ± 0.30	0.88 ± 0.10	0.88 ± 0.07	0.82 ± 0.21	Treatment × regions	1.73	0.181
SOD2 (relative expression)	Bread only	0.96 ± 0.11	1.51 ± 0.15	1.00 ± 0.10	0.92 ± 0.28	Treatment	0.31	0.736
	Bread + gold KF	1.09 ± 0.11	1.12 ± 0.15	0.87 ± 0.10	0.99 ± 0.28	Brain region	6.24	0.006
	Bread + green KF	1.11 ± 0.11	1.10 ± 0.15	0.92 ± 0.10	1.33 ± 0.28	Treatment × regions	0.91	0.516
AChE activity (nM H_2_O_2_/µg protein/min)	Bread only	45.97 ± 1.02	93.19 ± 1.01	39.86 ± 1.02	28.48 ± 1.04	Treatment	2.90	0.086
	Bread + gold KF	45.45 ± 1.02	93.20 ± 1.01	37.43 ± 1.02	24.96 ± 1.04	Brain region	3045.85	<0.001
	Bread + green KF	46.63 ± 1.02	93.55 ± 1.01	38.02 ± 1.02	26.02 ± 1.04	Treatment × regions	1.77	0.172

FRAP: ferric-reducing antioxidant potential (values that share the same letters in each region are not significantly different (*p* < 0.05)), ORAC: oxygen radical absorbance capacity, OPA: oxidative potential assay, CAT: catalase, SOD: superoxide dismutase, AChE: acetylcholinesterase. Representative Western blot figures of catalase, SOD1, and SOD2 are supplied in Supplementary Figures S1–S3.

## Data Availability

The data presented in this study are available on request from the corresponding author.

## Supplementary Materials

The following supporting information can be downloaded at: https://www.mdpi.com/article/10.3390/nu16081097/s1, Table S1: Plasma oxidative stress and antioxidant biomarkers. All values are presented as mean ± SE. Significantly different values within the same row are represented by different letters (ab; *p* < 0.05). Figure S1: Representative Western blot analysis of catalase and GAPDH expression in brain stem (**A**), corpus striatum (**B**), hippocampus (**C**), and prefrontal cortex (**D**) in a growing pig model in response to the bread, bread with gold kiwifruit (KF), and bread with green KF treatments. Figure S2: Representative Western blot analysis of SOD1 and GAPDH expression in brain stem (**A**), corpus striatum (**B**), hippocampus (**C**), and prefrontal cortex (**D**) in a growing pig model in response to the bread, bread with gold kiwifruit (KF), and bread with green KF treatments. Figure S3: Representative Western blot analysis of SOD2 and GAPDH expression in brain stem (**A**), corpus striatum (**B**), hippocampus (**C**), and prefrontal cortex (**D**) in a growing pig model in response to the bread, bread with gold kiwifruit (KF), and bread with green KF treatments.
