# Supplementary material for: Effects of Green and Gold Kiwifruit Varieties on Antioxidant Neuroprotective Potential in Pigs as a Model for Human Adults
Source: Nutrients. 2024 Apr 9;16(8):1097. doi: 10.3390/nu16081097 (PMC11055029; doi:10.3390/nu16081097)

Supplementary material

Table S1 Plasma oxidative stress and antioxidant biomarkers. All values are presented as mean ± SE. Significantly different values within the same row are represented by different letters (ab; p < 0.05).

**Plasma biomarkers**	Bread only (n = 6)	Bread + Gold KF (n = 6)	Bread + Green KF (n = 6)^	*F*	*p*
Oxidative stress markers					
MDA (uM)	32.6 ± 1.1	35.0 ± 2.5	25.6 ± 5.8	1.76	0.21
Protein carbonyl (uM)	21.5 ± 3.1	13.9 ± 2.7	17.0 ± 3.7	1.44	0.27
Antioxidant markers					
FRAP (uM/mg protein)	14.0 ± 3.1^{a}	20.8 ± 3.3^{ab}	25.9 ± 1.4$^{b\wedge}$	4.27	0.04
ORAC (uM/mg protein)	21.5 ± 1.0	18.3 ± 1.5	19.8 ± 1.2	1.52	0.25
OPA (%ΔFI5min)	4.4 ± 0.1^{a}	3.5 ± 0.1^{b}	3.6 ± 0.0$^{b\wedge}$	33.21	<0.01

MDA: Malondialdehyde, FRAP: Ferric reducing antioxidant potential, ORAC: Oxygen Radical Absorbance Capacity, OPA: Oxidative potential assay. ^An outlier weas detected with studentised residuals ≥ ±3, leaving these groups with an *n* = 5.

Figure S2 Representative western blot analysis of catalase and GAPDH expression in brain stem (A), corpus striatum (B), hippocampus (C) and prefrontal cortex (D) in a growing pig model in response to the bread, bread with gold kiwifruit (KF) and bread with green KF treatments.

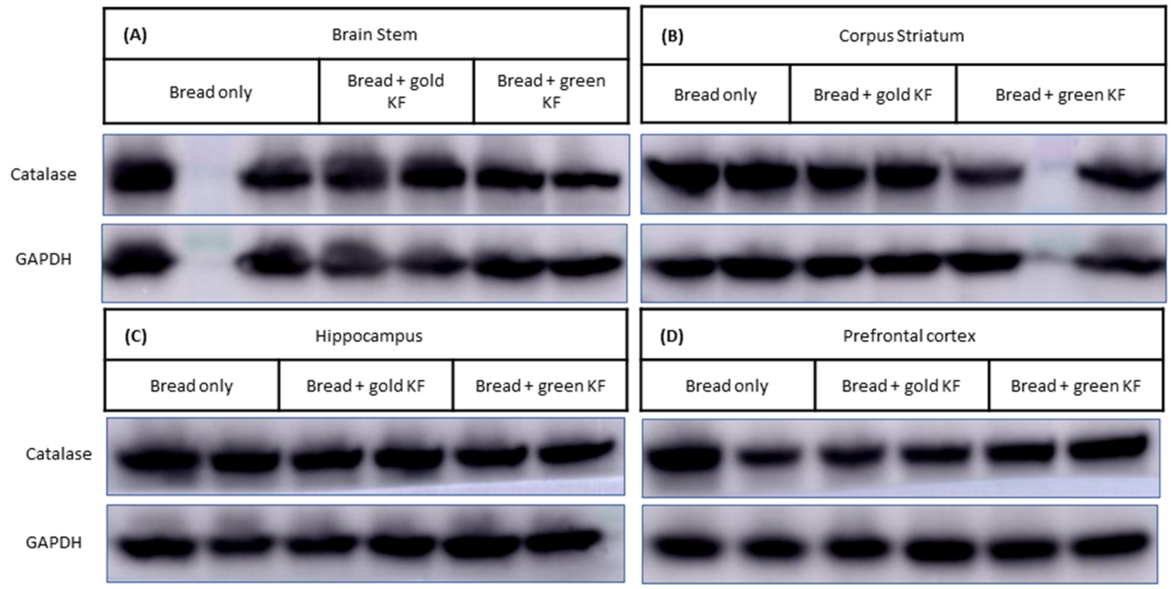

Figure S2 Representative western blot analysis of SOD1 and GAPDH expression in brain stem (A), corpus striatum (B), hippocampus (C) and prefrontal cortex (D) in a growing pig model in response to the bread, bread with gold kiwifruit (KF) and bread with green KF treatments.

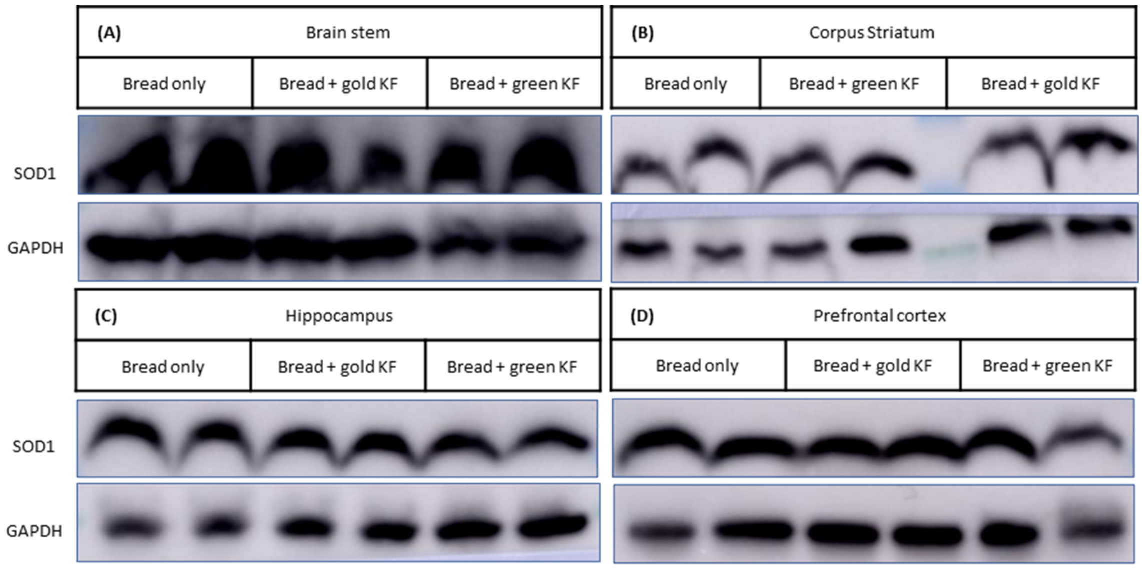

Figure S3 Representative western blot analysis of SOD2 and GAPDH expression in brain stem (A), corpus striatum (B), hippocampus (C) and prefrontal cortex (D) in a growing pig model in response to the bread, bread with gold kiwifruit (KF) and bread with green KF treatments.

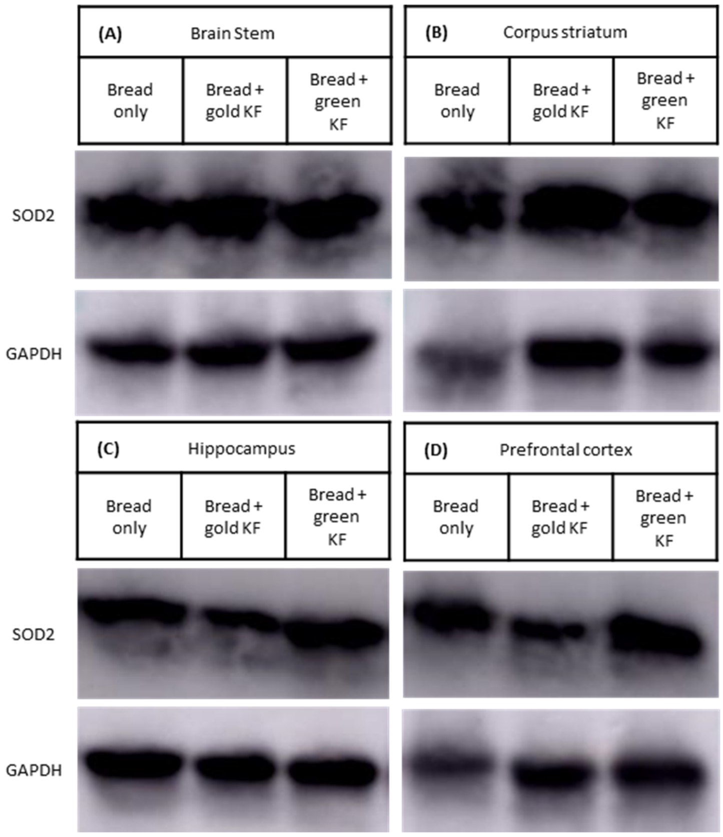

Supplement: Supplementary file 1 [file nutrients-16-01097-s001.zip › nutrients-2881186-supplementary.pdf]
